# Microalgae: Bioactive Composition, Health Benefits, Safety and Prospects as Potential High-Value Ingredients for the Functional Food Industry

**DOI:** 10.3390/foods11121744

**Published:** 2022-06-14

**Authors:** Josephine Ampofo, Lord Abbey

**Affiliations:** 1 Department of Food Science and Technology, University of California, Davis, CA 95616, USA; 2 Department of Plant, Food and Environmental Sciences, Dalhousie University, 50 Pictou Road, Truro, NS B2N 5E3, Canada; labbey@dal.ca

**Keywords:** microalgae, bioactive compounds, functional food, elicitation

## Abstract

Global population is estimated to reach about 9.22 billion by 2075. The increasing knowledge on the relationship between food biochemistry and positive health gives an indication of the urgency to exploit food resources that are not only sustainable but also impact human health beyond basic nutrition. A typical example of such novel food is microalgae, an aquatic microorganism with a plethora of diverse bioactive compounds including phenolics, carotenoids, vitamin B_12_ and peptides. Microalgal bioactive compounds have been shown to possess positive health effects such as antihypertensive, anti-obesity, antioxidative, anticancer and cardiovascular protection. Although, the utilization of microalgal biomass by the functional food industry has faced lots of challenges because of species diversity and variations in biomass and cultivation factors. Other documented challenges were ascribed to changes in functional structures during extraction and purification due to inefficient bio-processing techniques, inconclusive literature information on the bioavailability and safety of the microalgal bioactive compounds and the fishy odor and taste when applied in food formulations. In spite of these challenges, great opportunities exist to exploit their utilization for the development of functional foods. Microalgae are a renewable resource and have fast growth rate. Therefore, detailed research is needed to bridge these challenges to pave way for large-scale commercialization of microalgal-based healthy foods. The focus of this review is to discuss the potential of microalgae as natural ingredients for functional food development, factors limiting their acceptance and utilization in the food industry as well as their safety concerns with respect to human consumption.

## 1. Introduction

Functional role of foods has shifted from only the provision of energy and basic nutrients to include the supply of non-nutritive bioactive compounds capable of offering protection against the development of chronic diseases. Plant food groups (e.g., grains, legumes, nuts, fruits and vegetables, etc.) with these duo characteristics are defined as functional foods, which can be consumed as fresh foods, minimally processed or as ingredients in formulated foods [1]. However, with the increasing world population and consumer knowledge of the relationship between food intake and development of diseases, there is the need to exploit other food sources that can (i) sustain the functional food industry and (ii) meet the demanding health needs of the growing populace. In pursuit of this, an underexploited aquatic microorganism with potential functional food supply is microalgae.

**Figure 1:**
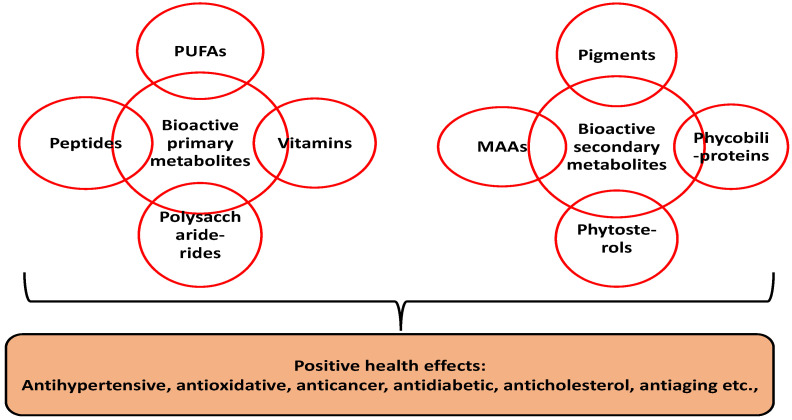
Bioactive composition of microalgal biomass. PUFA—polyunsaturated fatty acids; MAAs—mycosporine-like amino acids.

Compared to terrestrial plants, microalgae are sustainable due to their rapid growth, ease of cultivation and non-competition for arable land. Microalgae are unicellular aquatic microorganisms with over 50,000 classified species. Some notable examples include *Nostoc commune*, *Arthrospira platensis*, *Aphanizomenon flosaquae*, *Chlorella vulgaris* and *Chlorella pyrenoidosa* [2,3]. Besides their appreciable levels of primary metabolites (e.g., protein, carbohydrates, polyunsaturated fatty acids and vitamins), the health benefits of microalgae are mainly correlated to the presence of high value secondary metabolites. Secondary metabolites are non-nutritive compounds produced in plants as defense agents against environmental stress [4]. Microalgae research has shown substantial concentrations of diverse secondary metabolites such as pigments (e.g., phenolics, carotenoids, etc.), phytosterols and mycosporine-like amino acids [5] (Figure 1). 

**Figure 2:**
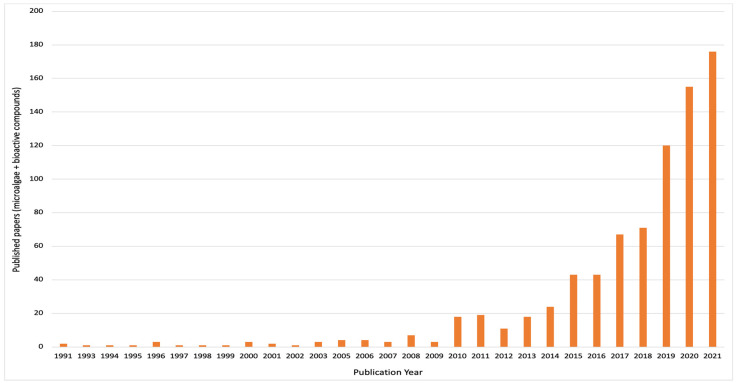
Bibliometric analysis on “microalgae and bioactive compounds” from the Web of Science database. The chart displays published research papers from January 1991 to December 2021.

Boussiba et al. [6] reported the total biomass of *Haematococcus pluvialis* to consist of 4% astaxanthin. In another study by Hifney et al. [7], phycobilin content of *Arthrospira platensis* was reported as 25% of its total dry biomass. According to literature, microalgal metabolites are of health value due to their capacity as antioxidant, antimicrobial, antitumor, anti-inflammatory and anticancer capacities [8,9]. Therefore, considering their renewability and plethora of high-value bioactive compounds, microalgae can be postulated as sustainable healthy food systems, which may be a reason for their increasing scientific literature over time (Figure 2).

The current review aim is to provide a comprehensive discussion on the microalgal composition of bioactive compounds, their biological activities, food applications, safety concerns and factors limiting their utilization in the functional food industry. This review further proposed innovative approaches to enhance the acceptance and utilization of microalgae as alternative plant-based novel food ingredients. 

## 2. High-Value Bioactive Primary Metabolites

### 2.1. Polyunsaturated Fatty Acids (PUFAs)

Polyunsaturated fatty acids (PUFAs) cannot be produced by the human body, hence, the need to obtain them through food consumption. They are divided into two groups namely omega-3 fatty acids (including α-linolenic, ALA; eicosapentaenoic acid, EPA; and docosahexaenoic acid, DHA) and omega-6 fatty acids (including arachidonic acid ARA; linoleic acid, LA; γ-linoleic acid, GLA; and conjugated linoleic acid, CLA) [10]. The health value of microalgae can partly be directed to its composition of PUFAs, which have been shown to promote brain and eye health, as well as protect against cardiovascular diseases, obesity, diabetes and arthritis [11]. Well-known PUFA microalgal producers include *Crypthecodinium*, *Schizochytrium* and *Ulkenia* sp. although other genera such as *Phaeodactylum*, *Monodus*, *Nannochloropsis* and *Porphyridium* have also shown considerable levels of DHA and EPA [12]. However, it is important to highlight that a majority of research data on microalgal PUFAs are reported with reference to their biofuel applications, with very limited reports on food, health and pharmaceutical applications. Considering the health benefits of PUFAs, coupled with the low consumer acceptance of fish oil PUFAs (i.e., low oxidative stability and high off-flavors), it can be postulated that there is the demand for alternative PUFAs. Hence, scientific data on microalgal PUFA profile, their bioactive properties and stabilities under different processing conditions will be very crucial in helping promote their applications in the functional food industry. In the study of Aussant et al. [13], eight species of microalgae were cultivated under different conditions (temperature—8, 14, 20 and 26 °C; time—5, 10 and 14 days). Of the eight investigated species, *Nannochloropsis oculata* and *Isochrysis galbana* reported the highest concentration of EPA (2.52 mg/L) and DHA (1.08 mg/L) at 20 °C/day 5 and 14 °C/day 5, respectively, with their investigated in vitro nutritional indices (i.e., hypocholesterolemic, atherogenic and thrombogenic indices) falling within accepted health ranges. Similarly, an increase in bicarbonate concentration from 2 to 8 mM increased the total PUFA content by 5.6% in *Pavlova lutheri* [14]. However, increasing light intensity from 37.7 to 100.0 µmol/m^2^/s in *Chlorella vulgaris* reduced DHA and EPA levels by 50 and 70%, respectively [15]. 

### 2.2. Polysaccharides 

From the review of Mourelle et al. [16], microalgal polysaccharides are largely exploited from the genera *Porphyridium*, *Phaeodactylum*, *Chlorella*, *Tetraselmis*, *Isochrysis* and *Rhodella*. In microalgae, polysaccharides function as protection agents, energy reservoirs and structural molecules, and are divided into pectins, glycol-protein, sulfated polysaccharides (SPS) and homo- and hetero-polysaccharides [17,18]. Among these polysaccharide groups, the most widely reported is the sulfated group with findings mainly reported on their anti-inflammatory benefits. In the study of Matsui et al. [19], extracts of sulfated polysaccharides from *Porphyridium* showed in vitro migratory inhibition of leukocytes to inflammation sites. These authors also observed in vivo microalgal inhibition against erythema development. Few antioxidant studies have also been reported with microalgae polysaccharides, as further discussed in Section 4 of the text. It is also important to highlight that microalgal polysaccharides are often exploited for their techno-functional applications, compared to their health benefits, thus, making it difficult to correlate their structure with health effects. Therefore, future studies focusing on the structure–activity relationship between microalgal polysaccharides and potential bioactive effects are highly recommended.

### 2.3. Vitamins

Although vitamins are essential elements required for proper human development, they can only be obtained through diets or supplements. Microalgae are excellent potential source of vitamins, compared to some well-known sources such as orange, carrot and soy flour [20]. Although microalgae are not natural producers of vitamin A, it is interesting to note that microalgae can accumulate vitamin A precursors such as carotenes (i.e., α- and β-carotenes) and retinol, which have been demonstrated to protect against the development of some cancer types [21]. The recent study of Ljubic et al. [22] investigated the accumulation of vitamin D_3_ (cholecalciferol) in *Nannochloropsis oceanica*, *Arthrospira maxima*, *Rhodomonas salina* and *Chlorella minutissima* upon exposure to different doses of ultraviolet B (0, 15, 22 and 36 kJ/m^2^/day) for 7 days. The authors observed the highest level of vitamin D_3_ (1 µ/g dry weight) with *Nannochloropsis oceanica* at UV-B dose of 36 kJ/m^2^/day, compared to the control (< 0.004 µ/g DW). Edelmann et al. [23] reported vitamin B_9_ contents in formulated powders of *Chlorella* sp. and *Nannochloropsis* sp. to be 25.9 and 20.8 µg/g, respectively. It can therefore be postulated that the consumption of about 5 g of *Chlorella* and *Nannochloropsis* microalgae powder can provide a quarter of the recommended daily intake (i.e., 400 µg/d) of vitamin B_9_. According to Tarento et al. [24], cyanobacteria has about 200 µg/g of vitamin K_1_, being about six times higher than levels reported for parsley (i.e., 37 µg/g), a well-known vitamin K_1_ food source. Hence, adult daily consumption of 1 g cyanobacteria will provide three times their daily needs for vitamin K_1_. Another crucial vitamin imperative for good health, especially among the aged is vitamin B_12_, although it is limited in plant foods [25]. Nevertheless, Edelmann et al. [23] observed *Chlorella* sp. to contain 2.4 µg/g of vitamin B_12_, concluding that 5 g *Chlorella* powder will provide five times the daily requirement of vitamin B_12_. A further important observation is that literature on bioaccessibility and bioavailability of microalgal vitamins is very limited. Thus, the need for deeper studies to enable health-regulating agencies and food industries to approve and include microalgae in the formulation of functional foods.

### 2.4. Peptides

Peptides are short chain amino acids (i.e., 20–50 units) linked together by peptide bonds [26]. According to Khanra et al. [27], 50% of the global protein and peptide market is currently sourced from terrestrial plants and may be replaced by proteins from microalgae and insects by 2054. Considering this trend, microalgal peptides has been exploited from *Chlorella*, *Navicula*, *Tetraselmis* and *Nitzschia* [2]. Ko et al. [26] isolated a pentapeptide with the amino acid sequence Leu-Asn-Gly-Asp-Val-Trp from *Chlorella ellipsiodea*, and reported appreciable peroxyl radical, 1,1-Diphenyl-2-picrylhydrazyl (DPPH) and hydroxyl radical scavenging capacities with half maximal inhibitory concentration values (IC_50_) of 0.02, 0.92 and 1.42 mM, respectively. Two isolated peptides from *Nannochloropsis oculata* with an amino acid sequence of Gly-Met-Asn-Asn-Leu-Thr-Pro and Leu-Glu-Gln were found to possess anti-hypertensive properties by inhibiting the activity of angiotensin-converting enzyme (ACE) at IC_50_ values of 123 and 173, respectively [28]. According to these authors, microalgal peptides can exhibit antihypertensive properties through the (i) inhibition of ACE, the main enzyme responsible for vasoconstriction of veins and arteries (ii) triggering of vasodilation effect, i.e., capacity to increase nitric oxide levels through the stimulation of the endothelial nitric oxide synthase pathway.

## 3. High-Value Microalgae Secondary Metabolites

### 3.1. Pigments

Pigments are secondary metabolites responsible for the coloring of living organisms, harvesting of light energy for photosynthesis and protection against oxidative stress. Up until now, the main groups of microalgal pigments include carotenoids, phenolics, chlorophyll and phycobiliproteins [2]. Details of the subgroups within these pigment groups are discussed further in the text, focusing mainly on their applications in functional food development. 

#### 3.1.1. Phenolic Compounds

Basically, phenolics are benzene ringed compounds, with hydroxyl groups present in their chemical structure. Depending on their carbon skeleton, phenolic compounds are divided into phenolic acids (e.g., hydroxybenzoic acids such as gallic and vanillic acids and hydroxycinnamic acids such as caffeic and coumaric acids), flavonoids (e.g., anthocyanin, quercetin and catechin), tannins (e.g., phlorotannins) and stilbenes [4]. Although microalgae have appreciable levels of phenolic compounds, majority of literature studies on aquatic phenolics are reported with macroalgae (i.e., seaweed). Nevertheless, the few literatures available on microalgae show significant variations due to specie type, cultivation conditions and techniques used for extraction, identification and quantification. *Chlorella pyrenoidosa* and *Arthrospira platensis* showed total phenolic levels of 25.8 and 43.2 mg/g, respectively [29]. In another study by Goiris et al. [30], phenolic composition of 32 microalgae species ranged between 54 and 375 mg/g. Additionally, Widowati et al. [31] observed total phenolic levels of 16.87 and 17.80 mg/g with *Tetraselmis chuii* and *Isochrysis galbana*, respectively. Interestingly, although literature has reported red grapes (*Vitis vinifera*) as the leading fruit producer of phenolic compounds, a study by Manach et al. [32] showed phenolic levels of microalgae to be comparable. However, to encourage their applications in the food industry, in-depth studies on the characterization of microalgal phenolic compounds are recommended to scientifically correlate their potential health benefits with specific functional foods. 

#### 3.1.2. Carotenoids

Carotenoids are lipid-soluble pigments mainly responsible for the purple, red, orange and yellow colors of living organisms. According to Rammuni et al. [33] about 600 carotenoid structures have been identified. Structurally, carotenoids are tetraterpenes consisting of eight isoprene units with a 40-carbon skeleton, divided into two groups namely carotenes and xanthophylls [34]. The carotene groups of lycopene and β-carotene consist solely of hydrocarbons (C_40_H_56_), whereas the xanthophylls (e.g., lutein, zeaxanthin and astaxanthin) are made up of carbon, hydrogen and oxygen (C_40_H_56_O_2_) [35]. Among microalgal species, the dominant carotenoid producing group is *Chlorophyceae*, which can yield about 90% of xanthophylls and carotenes with limited levels of fucoxanthin, diatoxanthin and diadinoxanthin [36]. Previous literature such as the study of Inbaraj et al. [37] investigated the presence of different carotenoids in *Chlorella pyrenoidosa*. Their study observed the presence of *cis* lutein isomers (27975.3 µg/g), *trans*-α-carotene (2465.8 µg/g), zeaxanthin (2170.3 µg/g), β-carotene *cis* isomers (2159.3 µg/g), *trans*-β-carotene (2155.0 µg/g), α-carotene *cis* isomers (1766.7 µg/g), β-cryptoxanthin (334.9 µg/g), neoxanthin and its *cis* isomers (199.7 µg/g), neochrome (65.2 µg/g), auroxanthin (38.5 µg/g) and violaxanthin and its *cis* isomers (38.1 µg/g).

##### β-Carotene

From the work of Khanra et al. [27], β-carotene is prevalently produced by *Scenedesmus almeriensis*, *Dunaliella bardawil*, *Dunaliella tertiolecta* and *Dunaliella salina*. Consumption of microalgae can help meet the physiological need of vitamin A, since research has reported the body’s capacity of converting β-carotene to vitamin A. Among microalgae species, *Dunaliella salina* has been reported as the highest source of β-carotene, exhibiting about 98.5% β-carotene in relation to its total carotenoid content (13%, dry biomass) [33]. Compared to other sources, microalgal β-carotene has been shown to be a rich source of 9-cis β-carotene (i.e., a cyclic carotene with the structure of β-carotene, but with a cis double bond attached to positions 9 and 10) [36]. In fact, this structure is a major contributor of its free radical scavenging capacities. Secondary metabolites of some microalgae are presented in Table 1.

##### Lutein

Lutein has gained much research interest in recent years due to its food coloring property and nutraceutical value, especially as antioxidant and anticancer agents, with intake up to 60 mg/day safe for adults of 60 kg body weight [53]. Previous research has correlated lutein with antioxidative, antiaging, anti-cardiovascular and anticancer benefits [9]. Among microalgae, the *Chlorella* genus is reported as the best source of lutein, with *Chlorella protothecoides* being dominant (about 4.6% on dry weight basis) [54]. Other microalgal species such as *Muriellopsis* sp. *D. salina*, *Scenedesmus almeriensis* and *Galdieria sulphuraria* are also exploited for lutein [55]. Although industrial exploitation of natural lutein is sourced from marigold petals (i.e., 0.03% on dry weight basis), lutein concentrations in *Chlorella protothecoides* and *Muriellopsis* sp. are 150 and 18 times higher compared to marigold [56]. 

##### Fucoxanthin

This compound accounts for about 10% of total carotenoids found in nature. It is abundant in microalgae when compared with other aquatic plants. Fucoxanthin is a lipophilic compound characterized by a carotene structure and an oxygenated backbone, with the presence of functional groups such as hydroxyl, epoxy, carboxyl and carbonyl moieties [57]. It absorbs light within ranges of 450–540 nm, thus translating light in the blue-green to yellow-green sections of the visible spectrum. Previous studies have reported positive bioactive benefits of fucoxanthin-rich foods such as antioxidant, antimicrobial, anticancer, antihypertensive and many others [58]. These bioactive benefits have been attributed to different mechanisms of action such as induction of apoptosis, cell cycle arrest, scavenging of intracellular reactive oxygen species and inhibition of cell proliferation [59].

##### Astaxanthin

Among the aquatic food chain, microalgae represent the major source of astaxanthin [60]. Astaxanthin, a red colored xanthophyll, is the second most exploited microalgae carotenoid after β-carotene [21]. Among microalgae species, the main producer of astaxanthin is *Haematococcus pluvialis*, with about 81% in relation to its total carotenoid yield of 7% on dry weight basis [2]. Besides *Haematococcus pluvialis*, species such as *Chlorella zofingiensis*, *Chlorococcum* sp. and *Scenedesmus* sp. can also accrue astaxanthin but at limited levels. Antioxidant capacity of astaxanthin is 10 times higher compared to lutein, β-carotene, lycopene and zeaxanthin and even 100 times higher than vitamins C and E [16,41], with this effectiveness attributed to its structural composition of thirteen conjugated double bonds. Considering their high antioxidant capacity, astaxanthin-rich microalgae can be exploited as novel ingredients for functional food development. Currently, this is being applied by the aquaculture industry, where astaxanthin rich microalgae are used as feed for fish and crustaceans [61]. For instance, Kim et al. [62] observed that the consumption of astaxanthin from *Haematococcus* sp. decreased lipid peroxidation and oxidative damage among heavy smokers. In another study, Yoshida et al. [63] fed 61 non-obese participants with an astaxanthin extract (i.e., AstaReal^®^) from *Haematococcus pluvalis* for a period of two weeks. After the study period, participants fed with astaxanthin formulated diets (i.e., 12–18 mg/day) showed increased serum HDL and adiponectin levels compared to those that were fed on placebo. 

##### Zeaxanthin

Zeaxanthin is predominantly produced by microalgal strains of *Scenedesmus almeriensis* and *Nannochloropsis oculata*. Research has proved a simultaneous relationship between zeaxanthin intake and health benefits such as protection against macular degeneration, cataracts, cancer and tumors [57]. Koyande et al. [21] associated visual health effects of zeaxanthin with the presence of nine conjugated double bonds and hydroxyl group in its chemical structure. This functional structure demonstrates its strong ability to absorb blue light and protect against retina damage. According to the findings of Ravi et al. [64], the daily consumption of zeaxanthin (i.e., 400 mg/kg body weight) showed no acute, sub-chronic toxicity or mutagenic effects. This is an attestation of the safety of zeaxanthin when consumed at recommended levels. It should also be added that zeaxanthin is not only beneficial for eye health but also protective against the development of certain cancer types (e.g., pancreatic and breast cancers) and type 2 diabetes [35].

#### 3.1.3. Chlorophyll

Depending on the microalgal strain and growth conditions, the chlorophyll content of a cell ranges from 0.5 to 4% of the total dry mass [21]. Chlorophyll is a natural green pigment crucial in photosynthetic organisms for harvesting of energy from sunlight. They exist as either chlorophyll *a*, *b* or *c*. However, chlorophyll *c* is only present in brown algae and not in green algae [21]. Species of *Chlorella* are the main producers of chlorophyll, with other species such as *Spirulina* and *Arthrospira* producing limited concentrations [27]. Besides their photosynthetic role, chlorophyll has been shown to exhibit strong antioxidant capacity. In the recent study of Suparmi et al. [65], sodium-nitrate-treated Wistar rats were fed chlorophyll extracts (8 and 16 µg/mL) from *Sanropus androgynus* for 14 days, after which the authors observed strong antioxidant protection in treated Wistar rats in in vivo studies. 

### 3.2. Phycobiliproteins 

Phycobiliproteins are colored hydrophilic proteins capable of absorbing light energy between wavelength ranges of 470–660 nm [45]. According to their level of light absorption spectra, phycobiliproteins are divided into four main groups including phycoerythrins (540–570 nm), phycocyanins (610–620 nm), allophycocyanins (650–6500 nm) and phycoerythrocyanins (560–600 nm) [44]. Phycobiliproteins are predominantly found among cyanobacteria (e.g., *Spirulina*), red algae (e.g., *Porphyridium and Galdieria*), cryptophyte and glaucophyte [66]. Structurally, they are composed of two dissimilar peptide chains covalently attached to chromophores. In fact, observations by previous researchers led to the conclusion that the amino acid composition of phycobiliproteins in combination with the presence of double bonds in their chromophores are primarily responsible for their wide range of antioxidative capacities [43]. Phycocyanin is a natural, blue-colored pigment applied in formulations of confectionary, dairy, snacks and beverages. They offer health benefits such as anti-inflammatory, antiviral, antioxidative, hepatoprotective and neuroprotective effects [43,67]. Indeed, the much-advocated health benefits from spirulina consumption have been widely attributed to its appreciable concentration of phycocyanin.

### 3.3. Mycosporine-like Amino Acids (MAAs)

Mycosporine-like amino acids (MAAs) exhibit UV absorbance capacities between 310 and 365 nm. Structurally, MAAs are low molecular weight (<400 Da) molecules composed of either cyclohexenone or cyclohexenimine chromophores conjugated with one or two amino acids [68]. Notable examples of MAAs include shinorine, palythenic acid, mycosporine-glycine, mycosporine-taurine, palythine and porphyra-334 [69]. Majority of MAAs data on health benefits are based on their UV-absorbing capacities, with very limited literature on food applications. However, taking advantage of their reported free radical scavenging capacities, the application of MAAs as natural food preservatives can be postulated. There is the need to investigate other bioactive effects of MAAs besides their UV-absorbing and free radical scavenging capacities in-order to increase their utilization in the food industry.

### 3.4. Phytosterols 

Phytosterols are plant-based steroids such as animal cholesterol but vary only in their carbon side chains and/or the presence of a double bond compared to animal cholesterol [70]. Phytosterols are made up of two different groups, namely sterols and stanols, with sterols possessing a double bond in its structure (i.e., making it unsaturated) and vice versa for the stanols (i.e., making it saturated) [70]. Recently, microalgal phytosterols are receiving attention from researchers due to their positive health impacts such as anticancer, antioxidative, anti-inflammatory, anti-atherogenicity, cardiovascular protection and reduction in LDL cholesterol [71].

Microalgae genera such as Dinoflagellates are key producers of 4α-methyl sterols, *Pelagophyceae* sp. are dense in 24-propylidenecholesterol, whereas *Glaucocystophyte* sp. are not only rich in sitosterols but also campesterol and stigmasterol [72]. Furthermore, Ahmed et al. [73] reported phytosterol production capacity in 10 microalgae species, with their work reporting *Pavlova lutheri* as the best producer of phytosterols (5186 mg/100 g) among other investigated species such as *Tetraselmis* sp. and *Nannochloropsis* sp. Thus, depicting the sustainable use of microalgae as a phytosterol resource compared to current options such as canola (*Brassica napus* L.) and corn (*Zea mays*) which produce about 820 and 850 mg phytosterol/100 g, respectively.

## 4. Bioavailability and Health Impacts of Microalgae as Food Ingredients

### 4.1. Antioxidative Properties

Because the human body is an active respiratory system, there is always the release of free radicals which can induce oxidative stress [21]. Therefore, to keep free radicals under control, the body protects itself through the actions of antioxidant enzymes (e.g., catalase and superoxide dismutase) or dietary antioxidants (e.g., pigments and peptides). Microalgae pigments such as phenolic compounds can exhibit antioxidant activities through their donation of hydrogen atoms to unstable free radicals [2]. Besides microalgae concentration of phenolic compounds, one important factor that contributes to their antioxidant capacity is the types of phenolic compounds present in its biomass. 

De Jesus Raposo et al. [74] correlated antioxidant capacity of carotenoids with the presence of conjugated double bonds and specific functional groups such as epoxy, acetyl, allene and acetylene. Kent et al. [75] observed that peptides from *Alexandrium minutum* activated targeted cell death in human lung cancer cell lines (Wi38) at an IC_50_ value of 0.7 µg/mL with no detrimental effect on normal cells. Ko et al. [26] isolated and cultured a pentapeptide (amino acid sequence of Leu-Asn-Gly-Asp-Val-Trp) from *Chlorella ellipsiodea* with monkey kidney cells exposed to hydrochloride cytotoxicity. From their results, the pentapeptide at concentrations of 25, 50 and 100 µM enhanced cellular viability against hydrochloride-induced cytotoxicity by 56.3, 72.3 and 79.4%, respectively. 

### 4.2. Hypertension

Currently, the drug lisinopril is applied for treatment of hypertension but has been linked with side effects such as dizziness, headache, vomiting and diarrhea [76]. Therefore, there is a need to explore natural alternatives. Ko et al. [26] observed a positive correlation between peptides from *Chlorella ellipsiodea* and the inhibition of the angiotensin-converting enzyme (ACE), the enzyme responsible for vasoconstriction of blood vessels. The investigated peptide (i.e., Val-Glu-Gly-Tyr amino acid sequence) showed ACE inhibition activity (IC_50_) of 128.4 µM. From this same study, the authors further observe that Md reduced systolic blood pressure effects when the investigated peptide fractions were fed to rats via in vivo studies.

### 4.3. Antimicrobial

A disc diffusion assay by Desbois et al. [77] showed that PUFAs (i.e., EPA, hexadecatrienoic and palmitoleic acids) from *Phaeodactylum tricornutum* exhibited high antibacterial activity against *Staphylococcus aureus*. Scaglioni et al. [78] also investigated antifungal and anti-mycotoxigenic potential of phenolic extracts from *Nannochloropsis* sp. and *Spirulina* sp. against cultures of *Fusarium graminearum* in trichothecenes mycotoxins. The authors observed that, 40 µg/mL of *Nannochloropsis* sp. phenolic extracts showed 98 and 100% inhibition of acetylates and nivalenol and deoxynivalenol, respectively. Additionally, phenolic extracts obtained from *Spirulina* sp. reduced the production of deoxynivalenol, acetylates and nivalenol by 62, 78 and 100%, respectively. 

### 4.4. Anti-Inflammation

A 100 µg/mL carotenoid extract comprising lutein, violaxanthin, antheraxanthin, neoxanthin and carotenes from *Tetraselmis suecica* showed anti-inflammatory effects in human cells exposed to 30 mM H_2_O_2_ oxidative injury [79]. Saini and Keum [80] also reviewed isolated polysaccharides from *Chlorella stigmatophora* and *Phaeodactylum tricornutum* and reported higher anti-inflammatory effects (i.e., IC_50_ 2.25 and 2.92 mg/kg, respectively) in rats exposed to carrageenan-induced paw oedema compared to the synthetic drug indomethacin (IC_50_ 8.58 mg/kg). Chrysolaminarin, a water soluble β-1,3-D-glucan isolated from the diatom *Synedra acus* exhibited anti-proliferative effects against human colon cancer cell lines DLD-1 and HCT-116, at IC_50_ values of 47.7 and 54.5 µg/mL, respectively [81]. Additionally, due to the vigorous antioxidant and microbiota regulation capacities of microalgal fucoxanthin, it is currently being applied for the treatment of various inflammatory-related diseases [82].

### 4.5. Anti-Proliferative and Cardiovascular Effects

A study by Murai et al. [83] which involved 86,113 participants (i.e., 40,707 men and 45,406 women) revealed an inverse relationship between seaweed/microalgae consumption and risk of ischemic heart disease. In another study, animal diets formulated with the polysaccharide-rich red algae, *Rhodella reticulata*, resulted in reduced serum cholesterol levels [84]. From this same study, animals fed with polysaccharide-rich red algae showed reduced levels of glucose and insulin. From the literature, it is notable that few studies have investigated the health benefits of microalgal-based bioactive compounds either in vivo or in vitro. Indeed, if researchers believe that the world population is increasing tremendously and requires sustainable healthy food systems, then this research gap with microalgal studies should be addressed with all seriousness. Almeida et al. [85] investigated the effect of fucoxanthin on cancerous cell lines such as the human bronchopulmonary carcinoma cell line NSCLC-N6, erythromyeloblastoid leukemia cell line K562 and human lymphoblastoid cell line TK6. According to their study, all investigated cell lines showed positive effects upon treatment with fucoxanthin. Similarly, in vivo studies with mice showed reduced tumor growths of primary effusion lymphoma, sarcomas and osteosarcoma when fed with microalgal-based fucoxanthin extracts [86]. 

## 5. Microalgae Utilization for Food Formulations

Formulation of functional foods from microalgal biomass is expected to increase in the near future as the global demand for healthy foods increases. As discussed by Nova et al. [3], the application of microalgae for food formulations has opened a new phase for the functional food industry, since a continuous search for innovative raw ingredients targeted for novel food developments exists. However, as discussed in the review of Nethravathy et al. [87], essential questions to be addressed towards the efficient formulation of microalgal-based food products include:

1. Interaction of microalgal biomass with other food matrix components;
2. Impact of microalgal addition on food sensory attributes, especially color and taste;
3. Textural and rheological changes in formulated food products upon microalgal formulation;
4. Changes and stability of microalgal compounds upon exposure to processing conditions.

**Figure 3:**
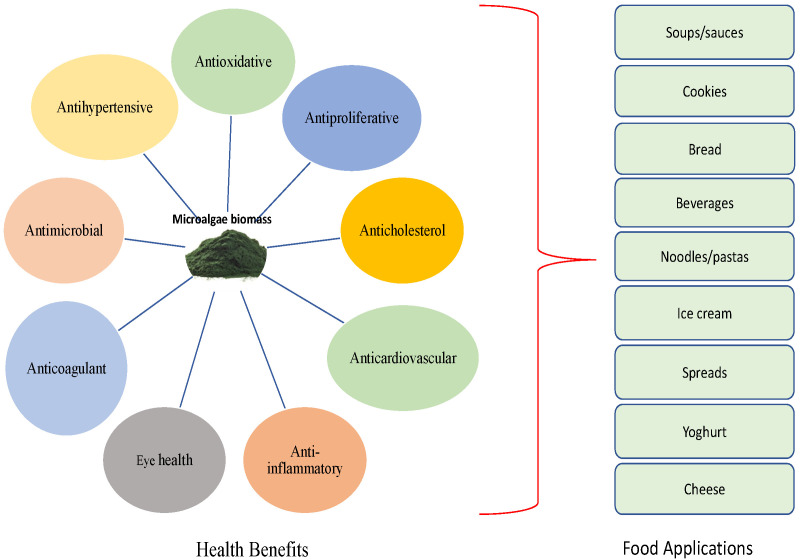
Schematic description of microalgae health benefits and potential food applications.

Development of eco-innovative techniques to meet these gaps will help maximize the utilization of microalgal-based ingredients in the current competitive functional food marketspace. Currently, the food industry is applying whole microalgal biomass or their extracted purified compounds as novel ingredients for the formulation of food products such as baked goods, pasta, noodles, plant-based milk, soups and many others, with a reflection of improved techno-functional and nutritional properties as depicted in Figure 3 and Table 2.

### 5.1. Snacks

**Figure 4:**
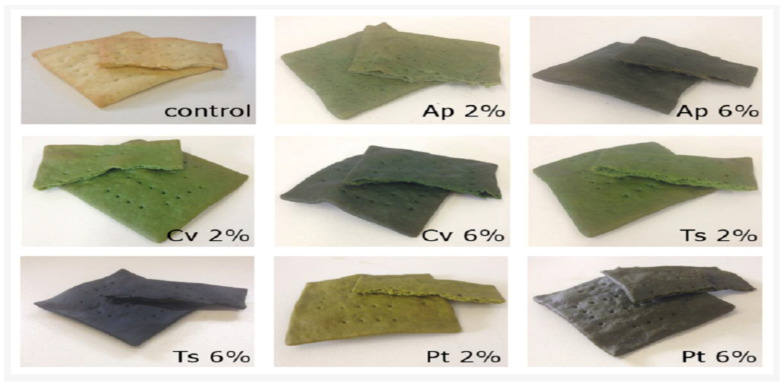
Snacks enriched with different substitution levels (2 and 6%) of microalgae biomass. Ap—*A. platensis*; Cv—*C. vulgaris*; Ts—*T. suecica*; Pt—*P. tricornutum*. Figure adapted from Batista et al. [90].

Due to the increasing busy lifestyle of modern-day consumers, there is a high demand for snacks that are not only cheap but also convenient and healthy. Lucas and his colleagues [94] formulated snacks with *A. platensis* (at 2.6%, *w*/*w*) and observed enhanced levels of protein, minerals and lipids at 22.6, 46.4 and 28.1%, respectively, compared to the control. The authors also observed a positive coloration of the developed snacks as impacted by *A. platensis* with no negative effect on its texture, flavor and taste, with an overall sensory acceptability of 82%. Similarly, Bolanho et al. [105] developed cookies from *A. platensis* at levels of 2 and 5% (*w*/*w*). According to their study, the highest formulation level of 5%, reported maximum levels of protein, fiber and ash compared to their 2 and 0% formulated cookie counterparts. However, from their sensory studies, cookies formulated with 5% *A. platensis* reported the lowest score for consumer acceptability. Thus, this evidence confirms the report of Nethravathy et al. [87] that a major challenge for the microalgae-based food industry is finding the right balance between microalgae formulation level, product health quality and final consumer acceptability. Without the proper answer to these gaps, the microalgae-based food industry will suffer major market drawbacks. In another study, cookies formulated with astaxanthin extract from *Haematococcus pluvialis* showed improved nutritional, antioxidant and phenolic profiles [106]. An example of a microalgae-formulated snack is displayed in Figure 4.

### 5.2. Bread

**Figure 5:**
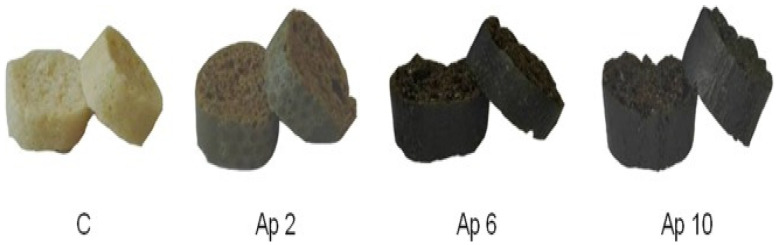
Sourdough crostini developed from *A. platensis F&M-C256* at formulation levels 2, 6 and 10%, *w*/*w*. C—control; Ap*—A. platensis*. Figure adapted from Niccolai et al. [106]; Source—Springer Nature; https://creativecommons.org/licenses/by/4.0/legalcode, accessed on 27 April 2022; no changes were made to the figure in re-printing.

Undoubtably, bread is one of the most widely consumed food products globally [107,108]. Based on the wide consumption and popularity of bread, developing functional bread will be a great vehicle for global food and nutrition security. In an interesting study by Uribe-Wandurraga et al. [108], breadsticks formulated with *Chlorella vulgaris* and *A. platensis* reported increased levels of iron and selenium, as well as color and texture stability over a storage period of 15 days, compared to their control forms. In another study, bread developed with 3 g of *Chlorella vulgaris* per 100 g of wheat flour showed improved viscoelastic and rheological properties, compared to the control and formulations above 3 g [109]. Hafsa et al. [110] formulated bread with *A. platensis* at 1 and 3% (*w*/*w*) substitution levels. From their study, although 3% formulation increased bread concentrations of minerals, protein and fat, this formulation level scored reduced consumer acceptability due to the green color impacted to the bread from microalgae. Thus, this evidence agrees with previous literature that the purchasing power of a food product also depends on its sensory attribute besides nutrition. Additionally, crostini formulated with different levels (0, 2, 6 and 10%, *w*/*w*) of *A. platensis* (Figure 5) showed increased protein, phycocyanin and total phenolic contents with increasing formulation levels, although this trend resulted in reduced in vitro digestibility [106]. Moreover, sensory analysis showed comparable acceptance for 2% microalgae formulated crostini with the control. Therefore, this suggests the strong need for a balance between microalgae food formulation, nutrition and consumer acceptability.

### 5.3. Others 

**Figure 6:**
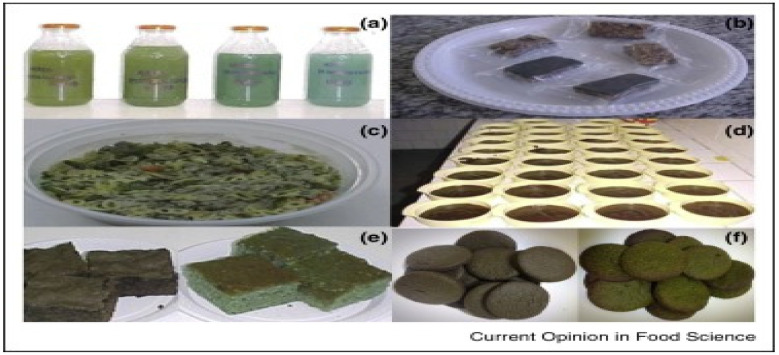
Different food formulations from *Spirulina* sp. ((**a**) isotonic beverages; (**b**) cereal bars; (**c**) instant soup; (**d**) pudding; (**e**) cake powder mix; (**f**) biscuit). Adapted with permission from [111], 2016, Elsevier Ltd.

Different food types formulated with *Spirulina* sp. are presented in Figure 6.

In one study, *Chlorella vulgaris* formulated with spreadable processed cheese at levels of 2, 4 and 6% (*w*/*w*) showed enhanced magnesium, potassium, selenium, zinc, iron and antioxidant potential, with this trend increasing with high formulation levels [112]. A functional yoghurt formulated with omega-3-rich lipid extract from *Pavlova lutheri* improved the concentration of n-3 PUFA for enhanced anti-inflammatory effects. However, although the study reported improved health benefits of the microalgae-formulated yoghurt, the authors also observed limited consumer acceptability upon sensory evaluation. In another detailed in vivo study, 1264.69 mg of omega-3-rich algal oil from *Schizochytrium* sp. was formulated with yoghurt and fed to 11 participants [113]. After 21 days of feeding, participants fed with formulated yoghurt significantly showed higher levels of bioavailable n-3-PUFA compared to those fed with unformulated yoghurt.

Consumption of soup is defined as a healthy dietary lifestyle due to its composition of different plant-based foods such as legumes, tubers and vegetables [3]. Lafarga et al. [114] developed broccoli soup formulated with 0.5 and 2% levels (*w*/*v*) of *Arthrospira*, *Chlorella* and *Tetraselmis* species. Upon simulated gastrointestinal digestion, the authors observed enhanced antioxidant capacity with high levels of bioaccessible polyphenols (i.e., between 32.9 and 45.6 mg/100 mL) for each microalgae/broccoli-formulated soup. Irrespective of the improved nutritional value, the authors observed reduced sensory acceptability with increasing levels of microalgae formulation. In contrast to this study, Los et al. [115] formulated a dehydrated soup with *A. platensis* at 15% (*w*/*w*), resulting in improved nutritional value (i.e., protein, fiber, chlorophyll, lipids and antioxidant capacity), with no compromise on acceptability when subjected to sensory evaluation. Based on these reports, there is a need for in-depth studies on formulation strategies that will enable effective microalgal food substitutions (i) for improved functionality and (ii) no/limited sacrifice of sensory quality.

## 6. Safety Concerns and Acceptability of Microalgae-Based Food Products

The effective contribution of microalgae to human nutrition has great potential. However, the acceptance and application of microalgae for functional food development will be greatly defined by their safety to human health. For instance, the FDA in United States has defined microalgal-based products as “other dietary supplements” with the consumption of strains such as *Spirulina*, *Dunaliella*, *Chlorella*, *Haematococcus*, *C. cohnii*, *P. cruentum* and *Schizochytrium* reported as GRAS. Compared to the United States, other countries such as Canada, Australia and New Zealand approve the consumption of limited microalgal strains including *Spirulina* and *Chlorella* [93]. These limitations are due to the dearth data available with respect to the safety and efficacy of microalgae-based food products/ingredients. However, the few available literature studies on different species of *Spirulina* and *Chlorella* have shown non-toxic concentrations of heavy metals, pesticides, antibiotics and mycotoxins. Notwithstanding, there is the need for continuous screening of dietary microalgal strains due to the reported presence of polycyclic aromatic hydrocarbons (PAH) above nutritional threshold levels [116]. Another possible factor for the low application of microalgae in the functional food industry may also be caused by the limited data on human allergic reactions. To increase their utilization and consumer acceptance, there is a need for in-depth research on microalgal allergic compounds, along with the demand of regulatory bodies to ensure food industries cite these possible effects on microalgal-based food labels. Additionally, some researchers and food processors argue on the dominating presence of pigments in different microalgal strains as a possible limiting factor in food applications, with reference to its possible negative effects on taste, color, flavor and other techno-functional properties. However, it can also be discussed that this is not always the case as the effect of a pigment will depend on the type of food product, as well as the group of consumers being considered as a target market. 

Another safety concern associated with food applications of microalgae is the high concentration of nucleic acid in its biomass, which has been linked to the negative effects of uric acid (i.e., responsible for gout and kidney stones) in consumers upon metabolism [87]. For example, toxins such as microcystin are elicited by *Microcystis aeruginosa*, anatoxin by *Anabaena flos-aquae* and saxitoxin by *Aphanizomenon flos-aquae*, with these toxic compounds associated with risks of gastrointestinal tract, liver, synapse and nerve axon functioning [117]. Thus, this provokes the conclusion that microalgae cultivation and processing when not conducted under safe conditions and continuous monitoring can lead to detrimental health. Overall, to ensure the safety and nutritional value of microalgal strains intended for human consumption, it is crucial for food industries to commercially cultivate microalgae under controlled environments compared to natural habitats such as lakes and ponds, since this approach can be less costly and also offer an efficient and consistent avenue for the industry to monitor key cultivation factors (e.g., water quality) which contribute to the possible accumulation of toxic compounds.

## 7. Challenges, Future Perspectives and Conclusions

With the predicted increase in global populations and climate changes, the future is faced with an unprecedented pressure for sustainable food resources capable of providing health benefits beyond basic nutrition. In this regard, microalgae are natural resources that can help meet this gap from the standpoint of sustainability, renewability and nutrition at a cheaper cost. However, some major obstacles concerning the utilization of microalgae as functional foods include accessibility, availability, sustainable techniques for extraction and purification of its metabolites, functional structure preservation during and after extraction and or in a multidimensional food matrix, bioavailability of extracted metabolites in the human gastrointestinal tract upon consumption, safety and sensory quality in formulated food products. 

Although genetic engineering has been employed to enhance microalgal composition of bioactive metabolites, there still exists a large proportion of the population with less acceptance and myths for genetically modified foods [118]. In this framework, less attention has been paid on how physical techniques (e.g., pulsed electric field and ultrasonication) and abiotic environmental factors (e.g., pH, temperature, and light) can be manipulated to improve microalgal yield of bioactive metabolites via the induction of oxidative stress, a biotechnological approach known as elicitation. This approach can be proposed as an all-inclusive consumer approach to providing bioactive-rich microalgal biomass. However, investigations on metabolic pathways associated with the production of microalgal bioactive metabolites are also needed, since they will help exploit elicitation techniques targeted for optimized yield of bioactive metabolites. Thus, opening the door for an economically efficient large-scale production and utilization of microalgae as novel sustainable food systems. 

With respect to the preservation of bioactive compounds from microalgal biomass during extraction and purification, there is the need to develop methods that cannot only optimize extraction yield but also protect the biochemical structures of less-stable bioactive compounds along the process, especially its functional hydroxyl groups. Another challenge concerning the functionality of microalgae-based bioactive compounds is the fact that a majority of their health benefits are reported with in vitro studies which mimic the human gastrointestinal tract and cannot be the same in reality. For instance, it has been well established that the passage of polyphenols, irrespective of the food matrix, in the stomach result in some being hydrolyzed, with about 90–95% passing into the large intestinal lumen [119]. Thus, it is very important to conduct clinical in vivo studies to not only elucidate the bioaccessibility, bioavailability and biological effects of their bioactive compounds, but to also confirm no side-effects upon consumption.

The pre-dominant sensory issues associated with microalgae-based food products are their intense color and fishy taste/aroma [120]. However, the food industry is taking advantage of the intense color, depending on the type of food product being developed and targeted consumers, as widely reported in the literature and many start-ups. Certainly, techniques such as nanotechnology and the use of safe molecules that act as inhibitors against bitter/astringent receptors are great avenues that can be exploited by the food industry for improved sensory acceptance of microalgae-based foods. Furthermore, it appears as if a majority of microalgae-based foods on the market are in the beverages, baked goods, and snack categories, with very limited application as pro/pre-biotics, although they have the potential for this purpose. A possible way to bridge this is the channeling of resources into the study of how microalgae grow and survive when being delivered alive through foods, as well as their functionalities in the human gut microbiota.

Furthermore, to increase the industrial utilization of microalgae for functional foods, there is a need to develop sustainable and eco-innovative processing techniques that are efficient for the transformation of raw microalgal biomass into value-added food products or isolated nutraceutical ingredients without compromising on their nutritional and environmental effects. With respect to the sustainable processing of microalgal biomass, novel non-thermal processing techniques such as fermentation, ultrasonication, pulsed electric field, microwave and enzyme-assisted processing can be very efficient in helping protect their nutritional value during processing while ensuring food safety concurrently. Lastly, with the increased wide choice of functional foods on the market, there is a need for effective collaboration between researchers and microalgal-based industries to better understand industrial loopholes and consumer needs. This will help guide the design of research outlines that will provide in-depth data on nutritional gaps, especially in vivo bioaccessibility, bioavailability and health benefits of functional compounds associated with microalgae consumption. Nevertheless, whatever the case, food industries should fully address rules and regulations governing the safety, quality and labelling of microalgal-based functional foods before commercialization.

## Figures and Tables

**Table 1:** Health benefits of secondary metabolites from selected microalgal species.

Genus/Species	Metabolite	Concentration	Bioactive Effect	References
*Dunaliella salina*	Lutein	0.4–0.8%	Antioxidative, anticancer, cataract and macular degeneration protection	[9,35]
*Chlorella sorokiniana*		5.21 mg/g		
*Heterochlorella luteoviridis*	β-carotene	0.19 mg/g	Anticancer, antioxidative, antihypertensive, neuroprotective, protection against macular degeneration, anticholesterol	[36,38]
*Dunaliella salina*		0.01–15.0 g/L		
*Chaetoceros* sp.	Fucoxanthin	1.5–2.0% 5.13 mg/g	Antioxidative, anticancer, anti-cholesterol, antidiabetic, antitumor	[39]
*Odontella* sp.				
*Chaetoceros* sp.				
*Haematococcus pluvialis*	Astaxanthin	1.95–2.75%	Antibacterial, anticancer, anti-inflammatory, antioxidative, neuroprotective, antimicrobial	[40]
*Chlorella zofingiensis*	Astaxanthin	1.5%	Antibacterial, anticancer, anti-inflammatory, antioxidative, neuroprotective, antimicrobial	[41]
*Heterochlorella luteoviridis*	Zeaxanthin	0.244 mg/g	Improve eye health, antidiabetic	[35,42,43,44,45,46]
*Dunaliella salina*		6 mg/g		
*Lyngbya* sp.		0.13 mg/g		
*Lyngbya* sp.		27.47 mg/g		
*Anabaena* sp. *Cyanidium caldarium*	Phycocyanin	8.3% 4–53 mg/g	Neuroprotective, anti-inflammatory, antioxidative, hepatoprotective, antiaging, antitumor	[47,48,49,50]
*Spirulina platensis* *Arthrospira platensis*		6.5–152 mg/g 54.65 mg/g		
*Oscillatoria quadripunctulata*		27.43 mg/g		
*Spirulina platensis*	Allophycocy-anins	26.8–45.2 mg/g	Hepatoprotective, antioxidative, anti-inflammatory	[47,51,52]
*Spirulina* sp. *Porphyridium cruentum*		20–70 mg/g 13.20 mg/g		
*Spirulina platensis*		92 mg/g		

**Table 2:** Nutritional and techno-functional properties of microalgae-based formulated food products.

Food Product	Genus/Species	Formulation Levels	Measured Effects	References
Biscuits	*Arthrospira platensis*	Whole *A. platensis* (0.3, 0.6 and 0.9%); phycocyanin extract: 0.3% *w*/*w* to wheat flour	Increased nutritional properties	[88]
Biscuits	*Arthrospira platensis*	1.63, 3, 5, 7 and 8.36% *w*/*w* to wheat flour	Improved techno-functional attributes and enhanced protein, fiber and antioxidant levels	[89]
Biscuits	*Arthrospira platensis*, *Chlorella vulgaris*, *Tetraselmis suecica* and *P. tricornutum*	2 and 6% *w*/*w* to wheat flour	Improved techno-functional and antioxidant properties	[90]
Cookies	*Haematococcus pluvialis*	Extracted astaxanthin powder at 5, 10 and 15% *w*/*w* to wheat flour	Enhanced techno-functional and antioxidant properties	[91]
Bread	*Arthrospira platensis*	11% *w*/*w* to flour	Improved techno-functional attributes, protein and mineral levels	[92]
Bread	*Isochrysis galbana*, *Tetraselmis suecica*, *S. almeriensis*, and *N. gaditana*	0.47% *w*/*w* to wheat flour	Increased techno-functional attributes	[93]
Extruded snacks	*Arthrospira* sp.	0.4, 1.0, 1.8, 2.6, and 3.2%, *w*/*w* to flour	Increased techno- functional attributes and protein levels	[94]
Butter cookies	*Chlorella vulgaris*	0.5, 1.0, 2.0 and 3.0%, *w*/*w* to wheat flour	Improved textural and color attributes	[95]
Biscuits	*Spirulina maxima*	5, 10 and 15%, *w*/*w* of wheat flour	Improved concentrations of protein, iron and PUFAs	[96]
Cookies	*Chlorella* sp.	3, 6, 9 and 12%, *w*/*w* of wheat flour	Improved techno-functional and sensory attributes	[97]
	*Spirulina platensis*	0, 1, 2 and 3%, *w*/*w* of wheat flour	Increased textural and colour properties; low overall sensory quality	[98]
Noodles	*Chlorella vulgaris* and *Spirulina platensis*	2.5, 5, 7.5, 10, 12.5, 15, 20 and 25 g, *w*/*w* of wheat flour	Enhanced techno-functionalities, protein, ash and dietary fiber; reduced carbohydrate content	[99]
Pasta	*Spirulina platensis*	5, 10 and 20 g, *w*/*w* to wheat flour	Enhanced techno-functionalities, protein, phenolics and antioxidant properties; limited protein digestibility	[100]
	*Dunaliella salina*	0, 1, 2 and 3%, *w*/*w* to wheat flour	Improved protein, minerals, antioxidants, techno-functional and sensory properties	[101]
	*Chlorella vulgaris* and *Spirulina maxima*	0.5, 1 and 2%, *w*/*w* of wheat flour	Elevated techno-functional and sensory qualities	[102]
Noodles	*Spirulina* sp.	3–5%, *w*/*w* of wheat flour	Improved nutritional quality	[103]
Protein drink	*Spirulina* sp.	10–12%	Improved protein content	[103]
Noodles	*Chlorella* sp.	-	Enhanced nutritional value	[103]
Energy bar	*Chlorella* sp.	5–10%	Enhanced protein content	[103]
Burger	*Chlorella* sp.	50%	Improved textural and sensory qualities	[104]

## Data Availability

Data are contained within the article.
